# Date Palm Extract (*Phoenix dactylifera*) PEGylated Nanoemulsion: Development, Optimization and Cytotoxicity Evaluation

**DOI:** 10.3390/plants10040735

**Published:** 2021-04-09

**Authors:** Hany Ezzat Khalil, Nashi K. Alqahtani, Hossam M. Darrag, Hairul-Islam Mohamed Ibrahim, Promise M. Emeka, Lorina I. Badger-Emeka, Katsuyoshi Matsunami, Tamer M. Shehata, Heba S. Elsewedy

**Affiliations:** 1 Department of Pharmaceutical Sciences, College of Clinical Pharmacy, King Faisal University, Al-Ahsa 31982, Saudi Arabia; pemeka@kfu.edu.sa (P.M.E.); tshehata@kfu.edu.sa (T.M.S.); helsewedy@kfu.edu.sa (H.S.E.); 2 Department of Pharmacognosy, Faculty of Pharmacy, Minia University, Minia 61519, Egypt; 3 Department of Food Science and technology, College of Agriculture, King Faisal University, Al-Ahsa 31982, Saudi Arabia; nalqahtani@kfu.edu.sa; 4 Research and Training Station, King Faisal University King Faisal University, Al-Ahsa 31982, Saudi Arabia; hdarag@kfu.edu.sa; 5 Pesticide Chemistry and Technology Department, Faculty of Agriculture, Alexandria University, El-Shatby 21545, Egypt; 6 Biological Sciences Department, College of Science, King Faisal University, Al-Ahsa 31982, Saudi Arabia; himohamed@kfu.edu.sa; 7 Department of Biomedical Sciences, College of Medicine, King Faisal University, Al-Ahsa 31982, Saudi Arabia; lbadgeremeka@kfu.edu.sa; 8 Department of Pharmacognosy, Graduate School of Biomedical & Health Sciences, Hiroshima University, 1-2-3 Kasumi, Minami-ku, Hiroshima 734-8553, Japan; matunami@hiroshima-u.ac.jp; 9 Department of Pharmaceutics, College of Pharmacy, Zagazig University, Zagazig 44519, Egypt

**Keywords:** *phoenix dactylifera*, nanoemulsion, date palm extract, optimization, cytotoxicity

## Abstract

Date palm fruit (*Phoenix dactylifera*) is reputed to have numerous biological activities, including anticancer properties. To utilize the great fortune of this fruit, the current study aimed to maximize its pharmacological activity. Date palm extract (DPE) of Khalas cultivar was obtained in powder form and then was formulated into nanoemulsion (NE). The optimized DPE-NE was formulated along with its naked counterpart followed by studying their physical and chemical properties. A qualitative assessment of total serum protein associated with the surface of formulations was implemented. Studies for the in vitro release of DPE from developed NE before and after incubation with serum were investigated. Eventually, an MTT assay was conducted. Total phenolic and flavonoid contents were 22.89 ± 0.013 mg GAE/g of dry DPE and 9.90 ± 0.03 mg QE/g of dry DPE, respectively. Homogenous NE formulations were attained with appropriate particle size and viscosity that could be administered intravenously. The optimized PEGylated NE exhibited a proper particle size, PDI, and zeta potential. Total serum protein adsorbed on PEG-NE surface was significantly low. The release of the drug through in vitro study was effectively extended for 24 h. Ultimately; PEGylated NE of DPE attained significant inhibition for cancer cell viability with IC_50_ values of 18.6 ± 2.4 and 13.5 ± 1.8 µg/mL for MCF-7 and HepG2 cell lines, respectively. PEGylated NE of DPE of Khalas cultivar will open the gate for future adjuvants for cancer therapy.

## 1. Introduction

As a fact, nature provides us with great reservoirs of plants as potential therapeutic candidates. Currently, the consumption of fruits and vegetables is considered important and beneficial for the health conditions to reduce the risk of several diseases such as cardiovascular disorders, aging, atherosclerosis, neurodegenerative conditions, and cancer. The valuable protective and curative activities are attributed in particular to their high dietary antioxidant contents, which can protect the human body against various oxidative reactions [1,2]. One of the most famous types of edible fruit is date fruit. Date fruits are cheap and rich source of carbohydrates, proteins, amino acids, and essential minerals (zinc, copper, selenium, potassium, calcium, magnesium, phosphorus, manganese, and iron), fiber, vitamins (C and E), carotenoids, fatty acids, polyphenols and flavonoids [3,4,5,6].

The date palm (*Phoenix dactylifera*) is commonly known in Arabian countries as Nakhel Al-tamr and in English as the date palm. It is a member of the family Arecaceae. Due to the extreme arid and semiarid conditions in Arabian Gulf countries, very few plants can grow. The date palm is well adapted to desert environments that are characterized by extreme temperatures and water deficiency [7]. The date palm tree is a medium to tall-sized plant, 3–15 m tall. It is cultivated for its edible sweet fruits [8,9]. The date palm is thoroughly associated in the culture of the people of Arabian countries. Its socioeconomic importance is not only due to its nutritive value but also for its economic importance [10]. Date fruits are considered as one of the integral parts of the Arabian diet, as an enormous source of nutrition and low-cost food [11].

In Saudi Arabia, the date palm is one of the pivotal fruit crops with an estimated 24 million palms, to produce approximately a 1.1 million ton of dates annually which represents roughly 15% of the world’s production. Worldwide, more than 3000 date cultivars are known [12]. Saudi Arabia has a rich diversity of date cultivars of which Khalas, Sheshi, and Reziz are well known, especially in the Eastern Province of the Kingdom [13,14,15,16,17]. Although date pits represent around 11–18% of date fruit, they are considered of low market value due to their hard texture and contamination by fungi or insects during the processing of date fruits [18,19]. Despite several reports demonstrating the presence of high amounts of phenolic and flavonoid constituents in both the fruits and the pits of date palm, the fruits (edible and safe) are easier and cheaper to deal with comparable to pits [4,18].

Cancer is a disease described by continuous uncontrolled propagation of abnormal cancer cells and regarded as one of the most prominent reasons that lead to death in the whole world [20]. Its treatment requires good approachability of the drug to the cancer cell. As a result, targeted drug delivery is regarded as a suggested methodology for cancer treatment strategy [21]. Meanwhile, the research of nanotechnology is acquiring a lot of interest recently [22], where the physical and chemical properties of active pharmaceutical ingredients or food additives could be tremendously changed through its formulation via nanotechnology.

The application of nanotechnology in targeted drug delivery systems for the treatment of cancer has increasingly been amended. Targeted drug delivery comprises three main components: the active agent, targeting moiety, and the drug carrier [23]. Nanocarrier encompasses several systems for targeting the drug such as nanoparticles, micelles, liposomes, and nanoemulsion (NE). Once the drug is encapsulated into the nanocarrier, its effectiveness is impressively improved [23].

NE is one of the contemporary nanoformulations that has attracted attention as it offers copious benefits over conventional emulsions owing to its nanoscale particle size that help in enhancing the bioavailability of incorporated ingredients in a way that makes them appropriate for applications. It is a colloidal system, formed when two immiscible liquids are mixed together using an appropriate surfactant to get a stable single phase [24]. This system is characterized by its ability to incorporate both hydrophilic and lipophilic drugs, being more stable, having great potential to deliver the drug to different organs in the body, in addition to the easiness of its manufacturing [25] However, for more precision and to enhance the product characteristics and the developing process, several approaches were applied for optimizing the fabricated formulation which is identified as the quality by design (QbD) concept [26].

QbD is an organized approach including various designs for optimizing and inspecting the relationship between the independent and the response variables obtained, which improves the efficiency of the study [27]. Response surface methodology (RSM), such as central composite design (CCD), is effective software that is based on mathematical and statistical techniques for studying the model and optimizes the manufacturing process [28]. Such modeling tools are dependent on the influence of several variables on the response and interpret this impact via generating mathematical equations and graphical form [29].

Consequently, the current study helps in finding an active natural candidate from the fruits of the carefully selected Khalas variety date palm and formulate the extract in the form of NEs. A twenty-three full factorial design was applied to develop DPE-NE formulations and study the effect of certain factors on their physicochemical properties. Overall, the best-selected formulation containing date palm extract was evaluated physically and chemically. Finally, the cytotoxicity of DPE and its optimized NE formulation was investigated against MCF-7 and HepG2 cell lines. 

## 2. Results and Discussion

DPE was successfully obtained and used to develop various homogenous PEGylated NE formulations. All developed preparations being stable without any indication of phase separation at 25 °C.

### 2.1. Estimation of Total Phenolic Content and Flavonoid Content

The estimation of total phenolic and flavonoid contents showed that both constituents are abundant in the fruits of Khalas variety date palm. The estimated quantity of total phenolic contents in DPE and DPE-NE were 22.89 ± 0.013 and 22.61 ± 0.005 mg GAE/g of dry DPE, respectively. The total flavonoid contents in DPE and DPE-NE were calculated to be 9.90 ± 0.03 and 9.49 ± 0.15 mg QE/g of dry DPE, respectively. The total phenolic contents of the Khalas variety date palm was similar to those found in the same cultivar (around 22 mg GAE/g of dry plant material) in the study of Alhaider et al. (2018) [13], while the total flavonoid contents were higher than those found in the same study (around 3 mg QE/g of dry plant material). Reports showed the total phenolic content of some Saudi date cultivars ranged from 122 to 247 mg GAE/100g dried extract [3], Moroccan date cultivar ranged from 171 to 353 mg GAE/100 g dried extract, Tunisian dates cultivar ranged from 199–576 mg GAE/100 g dried extract, and Omani dates cultivar with 231 mg GAE/100 gm of fresh fruit. Some previous investigations showed that the total flavonoid contents were estimated for Algerian cultivars (15–299 mg QE/100 g dried weight), Iranian cultivars (1–81 mg CE/100 g dried weight), and Tunisian cultivars (6–54 mg QE/100 g fresh weight) [6,30]. These variations and differences could be because of various parameters such as age, location, applied agricultural practices, climatic conditions, storage, or even genetic factors [3,4,5,6].

### 2.2. Determination of Drug-Excipient Compatibility Study (FTIR Characterization)

**Figure 1:**
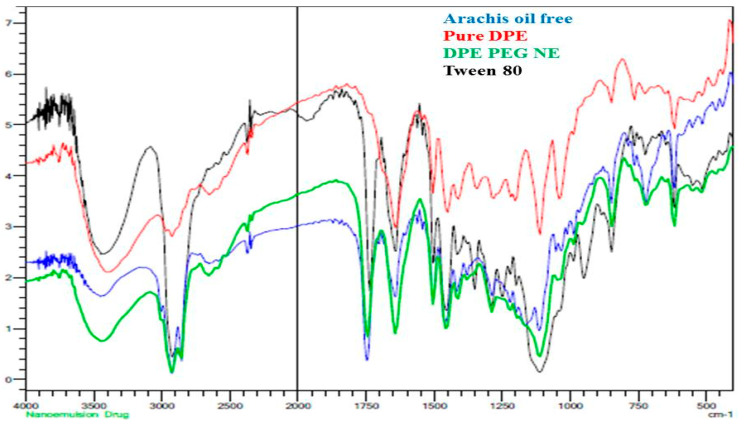
FTIR spectra of pure arachis oil, pure date palm extract (DPE), the developed PEG-nanoemulsion (NE), and tween 80.

The probability of interaction between DPE and the other substances utilized in the formulation manufacturing was inspected through Fourier transform infrared spectroscopy FTIR spectroscopy as noted in Figure 1. The spectrum of DPE showed a broad peak at 3400 cm^−1^ that could be related to the stretching vibration of hydroxyl groups of phenolic and polyphenolic constituents present in the DPE. Other peaks were displayed at 2900−3000 cm^−1^ that could be attributed to stretching vibration of C-H of aromatic skeletons like flavonoids or aromatic acids. A characteristic C=C stretching was found at 1650 cm^−1^ related to aromatics and other carbonyl C=O stretching of flavonoids or polyphenols. Other characteristic peaks related to stretching of C-C and bending vibration of C-H in aromatic rings at 1450 ~1500 cm^−1^, in addition to C-O group of polyols, such as hydroxy-flavonoids was around 1250 cm^−1^ [31]. Concerning the developed NE containing DPE, some peaks were around 1000–1150 cm^−1^ that would be related to secondary alcohols and/or to C-O- stretching ester group and vibration of C-C of -(CH2)n- side chains found in oil and emulsifying agents for the preparation of NE [32,33]. The additional peak at 1750 cm^−1^ representing the C=O of the ester group found in tween 80 and arachis oil. On the other side, the fingerprint region of DPE often existed while mixing it with other additives in PEGylated NE signifying that DPE included in the formulation had no significant chemical interaction.

### 2.3. Full Factorial Experimental Design

#### 2.3.1. Fitting the Model

The design matrix was produced by CCD software that ran 12 experiments including eight factorial points and four central points. In addition, the effect of independent variables on the response values were summarized in Table 1.

#### 2.3.2. Statistical Analysis of the Data

It is well known that when the *p*-value is lower than 0.05, the model is considered to be statistically significant. In the current study—as apparent in Table 2—, the *p*-values detected for the three responses, Y1, Y2, and Y3 were less than 0.0001 which confirmed the significant influence of the independent variables toward the examined responses without showing any chances of error [34]. Additionally, the greater F-values of the responses support the previous illustration where lower values revealed more error in the model. Further, values of the lack of fit help to inspect how the data is fitted to the model, thus, a non-significant lack of fit is requisite to fit the model [35]. As made clear in Table 2, the observed dependent responses showed the following values of lack of fit 5.34, 3.51, and 1.74 and *p*-values of 0.1011, 0.1651and 0.3299 for Y1, Y2, and Y3 respectively which established a non-significant lack of fit.

### 2.4. Viscosity Measurement

In regard to rheological behavior, data is displayed in Table 3. The formulated DPE-NEs exhibited a conventional viscosity hence it ranged between 2.38 ± 0.27 and 3.42 ± 0.23 cP depending on the concentration of ingredients included mainly oil and surfactant content. In fact, the results were in a proper range that could permit intravenous administration as formerly declared by Araújo et al., who estimated that it is reasonable for intravenous administration if the viscosity is lower than 3.9 cP [36]. These outcomes were in accordance with Elsewedy et al., who assessed the viscosity of Brucine NE to be up to 3.21 cP and established that it could safely be administered via intravenous route [37]. Further, Harun et al., estimated the viscosity of cefuroxime NE to be 1.24 cP and stated that it is acceptable for parenteral delivery as it is below 3.9 cP [38].

### 2.5. Size and Size Distribution (PDI) Determination

Particle size and PDI of the developed DPE-NE preparations were estimated and the results were displayed in Table 1. The particle size of all NE formulations was within the nanoscale and varying from 115 ± 1.5 to 235 ± 2.5, with PDI 0.24 and 0.452, respectively. These findings indicate that the distribution of the particle size is inside a narrow range which is good evidence for formulation stability. Truthfully, particle size and PDI are essential variables for the formulations administered intravenously since large particle sizes are clinically undesirable because of emboli formation that may happen (Araújo et al.). As well, it was previously revealed that formulations with PDI values smaller than 0.7 are considered perfect and confirm the homogeneity of the particle size in the preparation Danaei et al. [39]. It was observable that, increasing oil concentration from 1 to 2 g results in a subsequent increase in the particle size which could be ascribed to the increase in the dispersed phase [40]. Conversely, increasing surfactant concentration could reduce the particle size. The action of these factors on the observed response could be illustrated by the following regression equation:

Y_1_ = 167.5 − 9.66 A + 40.96 B − 2 AB − 1.43 A^2^ + 4.81 B^2^ (1)

**Figure 2:**
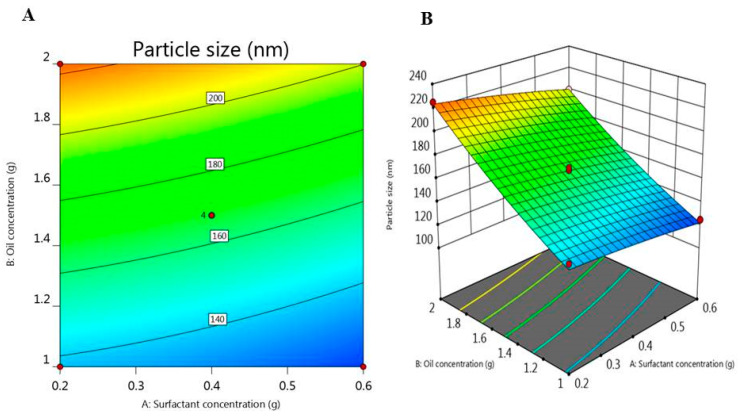
(**A**) 2D-Contour plot and (**B**) 3D-response surface plot representing the influence of surfactant concentration (g) and oil concentration (g) on particle size (nm).

Referring to the regression equation, the positive sign points to a synergistic effect however, the negative one denotes antagonistic action [27]. In the present equation, it was stated that variable (A) signified a negative influence on the particle size, though; variable (B) deliberated a positive upshot. Moreover, Figure 2A,B displayed a 2D Contour and 3D-response surface plot which exposed a non-significant effect of surfactant concentration (A) upon the particle size (Y1) of DPE-NE formulations however a substantial effect of oil concentration (B) was depicted.

### 2.6. Studies of DPE In Vitro Release from NE Formulations

**Figure 3:**
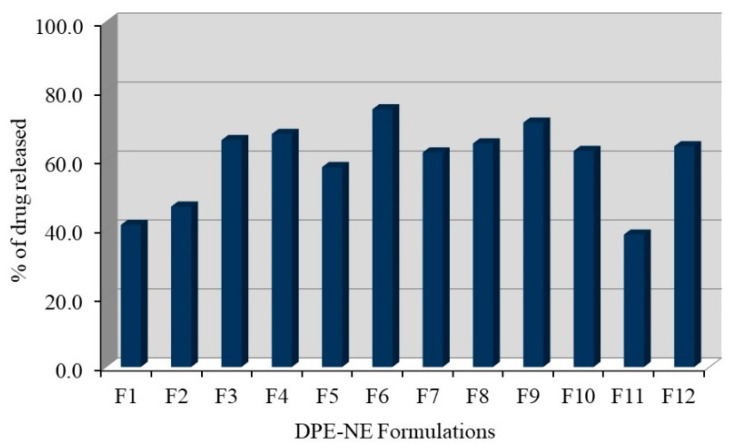
Representing DPE in vitro Release study from different NE formulations in phosphate buffer pH 7.4 at 37 °C. Results are stated as the mean ± SD of three experiments.

The in vitro release pattern of DPE-NE was carried out successfully and the release profile is plotted in Figure 3. It is evident from the figure that, following 24 h, the percentage of DPE released from formulated NE was (38.4 ± 6.5 and 74.7 ± 6.8%) from F11 and F6 respectively. Actually, decreasing oil concentration would result in a relative decrease in the particle size that offers a large surface area which in turn provides a higher percentage of drug released [41]. On the other hand, increasing surfactant concentration could increase the drug solubility in the aqueous media that resulted in improving the drug release [42].

**Figure 4:**
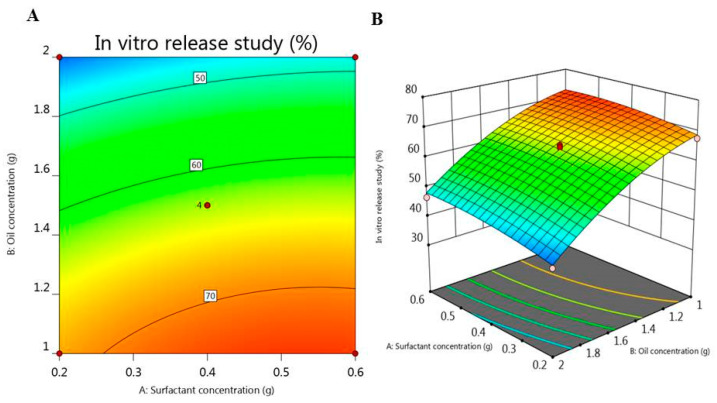
(**A**) 2D-Contour plot and (**B**) 3D-response surface plot representing the effect of surfactant concentration (g) and oil concentration (g) on in vitro release study (%).

Furthermore, the 2D-Contour and 3D-response surface plot portrayed in Figure 4, illustrates the effect of the independent variables on the investigated response (Y_2_). It was detected that surfactant concentration (A) exerted a significant synergistic effect on the amount of DPE released (Y_2_). Nevertheless, oil concentration (B) possessed an antagonistic effect (*p* < 0.05) on the in vitro release study. The fitted mathematical regression equation that describes the interaction of these independent variables and their considerable effects on the responses is given below:

Y_2_ = 63.4 + 2.44 A − 12.75 B + 0.52 AB − 1.42 A^2^ − 4.1 B^2^ (2)

### 2.7. Hemolytic Activity

**Figure 5:**
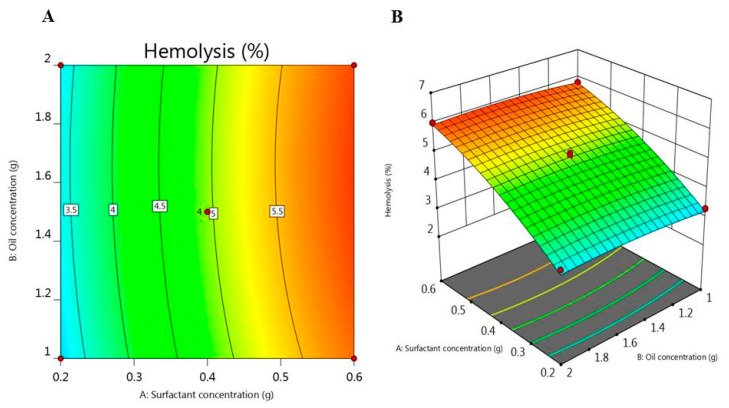
(**A**) 2D-Contour plot and (**B**) 3D-response surface plot representing the influence of surfactant concentration (g) and oil concentration (g) on hemolysis (%).

The hemolytic activity of the fabricated NE preparations was estimated in order to validate whether the formulations may come up with harmful effects on the biological membranes [43], and the data were demonstrated in Table 1. The percentage of hemolysis following incubation for 30 min was in the range of 2.4 and 6.2% in all formulations proposing that these preparations are harmless and suitable for intravenous administration. Likewise, as revealed in Figure 5, the surfactant concentration (A) employed a significant positive effect on the % of hemolytic activity (Y_3_), hence increasing surfactant concentration would result in a relative increase in the hemolytic activity of the preparations. Yet, oil concentration (B) did not greatly affect this response. The regression equation for hemolysis response (Y_3_) attained from response surface methodology is stated in the following equation:

Y_3_ = 4.95 + 1.29 A + 0.067 B + 5.15 AB − 0.28 A^2^ ± 0.10 B^2^ (3)

### 2.8. Optimization of Independent Variables

The objectives of the optimization process were to select the optimal characteristics and appropriate levels of constraints (Table 4) to get a formulation with proper features based on the higher desirability value [44]. The optimized DPE-NE formulation was selected depending on targeting the particle size to a range of 140 to 160 nm and in vitro release study to a range of 55 to 65% and minimizing the percentage of hemolysis.

**Figure 6:**
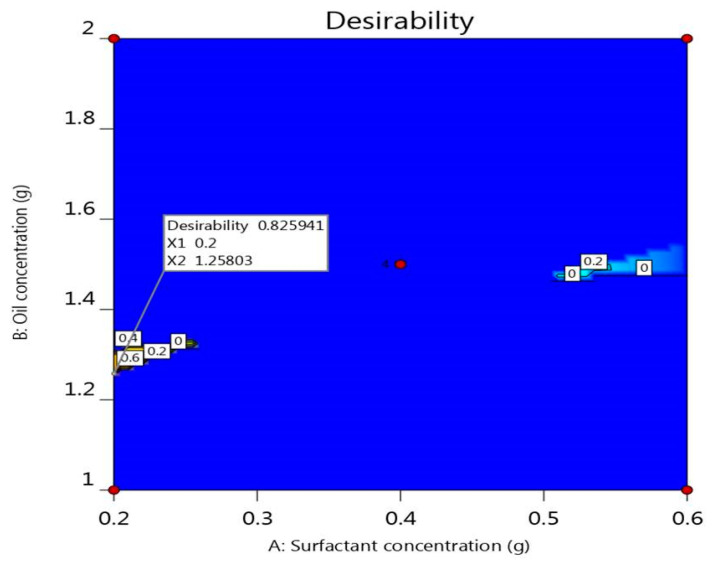
Optimization figure screening the influence of surfactant concentration and oil concentration on overall desirability.

The predicted values for the optimized DPE-NE formulation assumed by the software were displayed in Table 5, in addition to Figure 6 that suggests the higher desirability value (0.826). CCD predicted that the independent variables used in formulating the optimized preparation were 0.2 mg surfactant and 1.258 mg oil. The optimized NE formulation was then developed using the predicted values and was compared to the observed experimental values of the optimized formulation recorded in Table 5. Ultimately, the values of the experimental responses were in accordance with that of the predicted response.

Based on the previous investigations, a new formula was developed (PEG-NE) along with its naked counterpart (NNE), and both were subjected to certain further studies.

### 2.9. Particle Size and Zeta Potential Assessment

**Figure 7:**
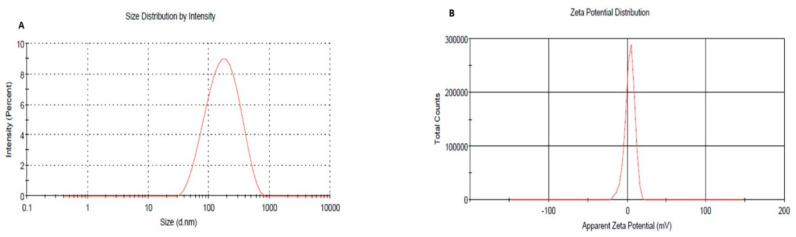

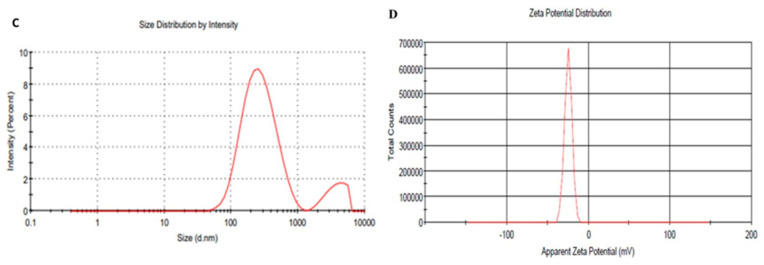
(**A**) Particle size of optimized PEG-NE; (**B**) Zeta potential of optimized PEG-NE (**C**) Particle size of naked-NE; (**D**) Zeta potential of naked-NE.

The measurement of particle size and estimating zeta potential of optimized formulation and its naked counterpart was determined and the results are shown in Figure 7A,B. It was detected that (NNE) showed a particle size 260 ± 5.5 nm and (PDI) 0.22 which is regarded as a higher value if compared to that of PEG-NE that recorded particle size 159.33 ± 3.4 with (PDI) 0.25 as presented in Figure 7C,D. This finding could suggest the upshot of PEGylation on particle size and the homogeneity of the NE formulation whereas uniformity of particle size has a great impact on the therapeutic efficiency of the formulation [45]. Regarding zeta potential, PEGylated NEs demonstrated a decline in the negative charge of the formulation surface (3.23 ± 0.26) compared to their naked counterpart (−24.6 ± 0.51) which is ascribed to the coating of the surface by PEG [46].

### 2.10. Determination of Serum Protein Associated on the Surface of NE Quantitatively

**Figure 8:**
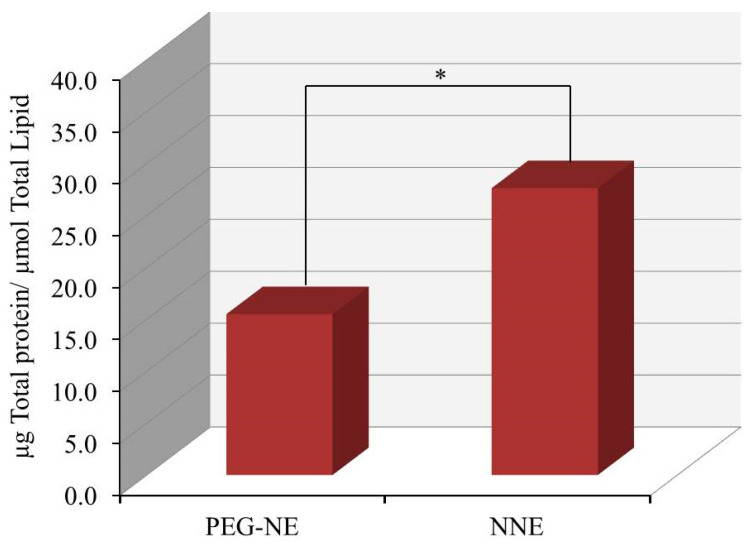
Total quantity of serum proteins adsorbed on NNE and PEG-NE surface. Results are stated as the mean with the bar showing S.D. (*n* = 3). * *p* < 0.05 if compared to naked counterpart.

The quantity of serum protein attached to the PEG-NE and its counterpart surface was measured and data is obvious in Figure 8. PEG-NE exhibited a significant minor quantity of serum protein attached on its surface (15.33 ± 2.08 µg/µmol total lipid) which is significantly different from the quantity adsorbed on its naked counterpart (NNE) surface (27.6 ± 2.3 µg/µmol) (*p* < 0.05). It could be established that the inferior quantity of serum protein detected on the surface of PEG-NE was ascribed to the PEG coat that resulted in a fixed aqueous layer thickness (FALT) phenomenon. FALT would have the capability to protect the formulation from being recognized by serum proteins and avoid its interaction with the PEG-NE outer surface [47]. In point of fact, the quantity of protein associated on the surface of NNE was expected to be higher than the recorded value. This is actually ascribed to the presence of tween 80 in the preparation which has a hydrophilic ethylene head group, that is likewise found in PEG-DSPE. This actively resists protein surface coatings, thus it could exhibit similar activity to PEG-DSPE [48].

### 2.11. Studies of DPE In Vitro Release from Optimized NE (before and after Serum Incubation)

**Figure 9:**
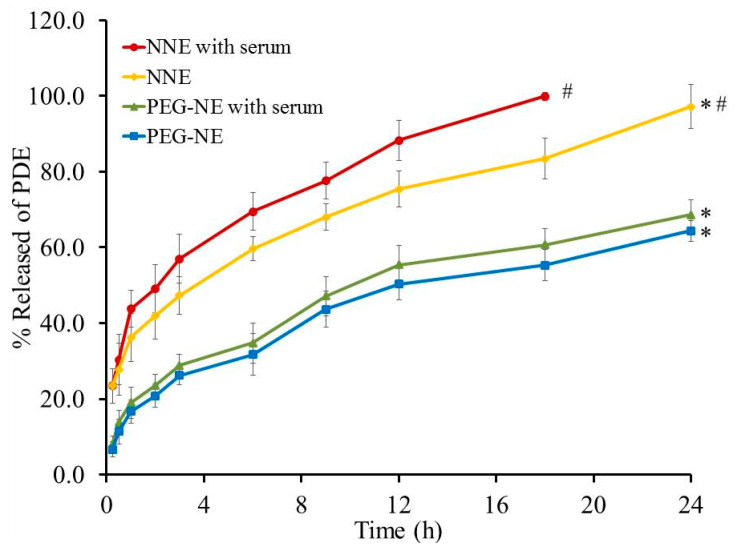
Study of DPE in vitro release from NE formulations before and after serum incubation. Results are stated as the mean with the bar showing S.D. (*n* = 3). * *p* < 0.05 if compared to NNE with serum. # *p* < 0.05 if compared to PEG-NE with serum.

The in vitro drug release pattern of DPE from PEG-NE formulation and its naked counterpart was accomplished and the profile was graphically plotted in Figure 9. Following 24 h, the study denotes that PEG-NE formulation showed (64.3 ± 2.7%) of DPE released compared to that released from NNE (97.17 ± 5.79%). It is manifested that the amount of the drug released from PEG-NE was significantly lower than that released from NNE. This actually could be owed to the good stability of PEGylated NE, in addition to the rigidization of the PEG-NE surface that resulted in a diminution of drug release [49].

On the other side, following incubating NE formulations with an equivalent amount of 10% serum, the release of the drug was estimated over 24 h and the effect is explained in the same figure (Figure 9). It was evident that serum accelerates the release of the drug from NNE as it reached 99.9 ± 1.1% over 18 h. Contrariwise, serum failed to affect in vitro release from PEG-NE since it achieved 68.6 ± 3.9% after 24 h compared to that without serum incubation (64.33 ± 2.7%). In view of that, there is an insignificant difference between in vitro release from PEG-NE before and after serum incubation which confirms the crucial role of PEG. The boosted release of drug from NNE could be elucidated to the statement of MAC assembly (membrane attack complex), which act to form lytic holes and permeable areas at the surface of the target surface [50]. Correspondingly, it was declared by Shehata et al. [51] that naked niosome subjected to attack by serum protein gave rise to formulation distraction leading to a higher amount of Doxorubicin released. Currently, about 66% of the running clinical trials are focused on cancer treatment utilizing nanotechnology. In order to be successfully introduced to the market, both FDA and EMA necessitate that nanoformulations must have maximum safety, efficacy, and quality criteria applied to final products [52]. The quality of nanoformulations includes physical evaluation (particle size, size distribution, and surface charge), in addition to the drug loading, in vitro drug release profile, and size stability during storage and after contacting the biological solutions [53].

### 2.12. In Vitro Cytotoxicity

**Figure 10:**
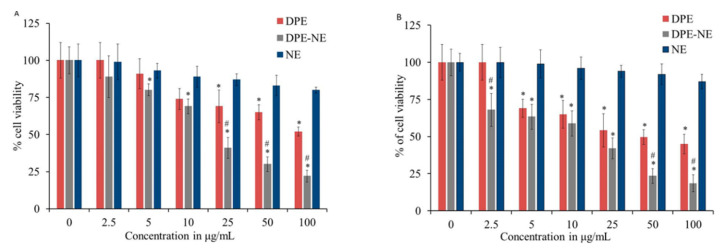
Cell viability evaluation of free DPE, optimized PEGylated DPE, and blank NE against (**A**) MCF-7 and (**B**) HepG2 cell lines following 48 h incubation. Results are stated as the mean ± S.D * Statistically significant with blank NE (*p* < 0.05). # Statistically significant with free drug (DPE) (*p* < 0.05).

The MTT colorimetric assay was carried out to evaluate cell viability of DPE PEG-NE against MCF-7 and HepG2 cells and the results were depicted in Figure 10. It is perceptible that a significant decline in the cell proliferation for PEG-NE was distinguished along with relevant IC50 values of 18.6 ± 2.4 and 13.5 ± 1.8 µg/mL for MCF-7 and HepG2 cells respectively if compared to free DPE that exhibited IC50 value of 101.3 ± 6.2 µg/mL on MCF-7 cells and 48.3 ± 4.4 µg/mL for HepG2 cells (*p* < 0.05). It was remarkable that incorporation of DPE within NE formulation stimulated cytotoxic activity that is higher than the free DPE [54]. As well, a significant diminution was detected in cell viability amongst PEG-NE and its corresponding placebo NE (*p* < 0.05). Remarkably, it was identified that there was a significant difference between free drug and placebo NE while using greater concentrations (25, 50, and 100 µg/mL) in the case of MCF-7. However, a significant difference was detected between them upon using lower concentrations (5 up to 100 µg/mL) in regard to HepG2 cells, which could be owing to the composition of the NE itself. Comprehensively, dealing with PEG-NE incorporating DPE exhibited a significant cell viability reduction in a dose-dependent manner, which could be attributed to increasing the drug solubility and boosting its cellular uptake once prepared in NE form [55].

## 3. Material and Methods

### 3.1. Material

Arachis oil and tween 80 were obtained from Sigma Aldrich (St. Louis, MO, USA). Distearoyl phosphatidylethanolamine-N-(methoxy poly (ethylene glycol)-2000) (PEG-DSPE) was purchased from Lipoid LLC, (Newark, NJ, USA). Fetal bovine serum (FBS) was obtained from Sigma Aldrich (St. Louis, MO, USA). Total protein and Total Lipid Colorimetric kits purchased from United Diagnostics Industry, (Dammam, Saudi Arabia). Tetrazolium dye (MTT reagent) was procured from Loba Chemie (Mumbai, India). All other reagents were of the finest grade available.

### 3.2. Attainment of Date Palm Extract (DPE)

#### 3.2.1. Dates Identification and Collection

Fruits of Khalas cultivar date palm were purchased from the local markets in Al-Ahsa, Eastern Province of Saudi Arabia. The date palm fruits were collected in the palatable stage and identified by experts and taxonomists in the date and palm center, King Faisal University, Al-Ahsa, Saudi Arabia. A voucher specimen of fruit Khalas date palm is deposited in the Herbarium of Department of Pharmaceutical Sciences, College of Clinical Pharmacy, King Faisal University, Al-Ahsa, Saudi Arabia (20-Sept-KH).

#### 3.2.2. Crude Extract Preparation

A weight of 250 g of dried flesh of fruits of Khalas date palm was macerated in 2.5 L of 70% methanol in distilled water applying cold maceration technique (1:10 ratio) for 48 h then filtered using the Whatman filter paper No.1. Methanol was selected as a solvent for extraction because of its power to extract all the various components from the plant material [56,57]. The process of extraction was repeated three times. The compiled extracts from the triplicate extraction process were then concentrated under reduced pressure and the remaining concentrated extract was freeze-dried to give the dried date palm methanol extract (DPE) and then was kept in dried condition for further experiments.

#### 3.2.3. Estimation of Total Phenolic Content

The total phenolic content of DPE was determined according to the method previously reported by Hany et al. [58]. Folin-Ciocalteu reagent protocol was applied in the assay of total phenolic content using gallic acid as the standard. To estimate the total phenolic content in DPE-NE, equivalent to 1mg of DPE in NE preparation was diluted in 3 mL of distilled water and stirred on a magnetic stirrer for 1 hour followed by 30 s of sonication. The resultant solution was cooled to room temperature and filter through 0.22 micro-syringe filters to give a clear solution. The concentration of phenolic contents was expressed as gallic acid equivalent (GAE) for 1 g of dried extract.

#### 3.2.4. Estimation of Total Flavonoid Content

The total flavonoid content of DPE was determined according to the protocol reported by Hany et al. [59]. Aluminum chloride (2% *v/v*) assay was implemented in the assay of total flavonoid content, quercetin was used as a standard. A stock solution prepared for the estimation of total phenolic contents in DPE-NE was used to estimate the total flavonoid content. The concentration of the total flavonoids was expressed as mg quercetin equivalent (QE) for 1 g of the dried extract.

### 3.3. Drug-Excipient Compatibility Study (FTIR Characterization)

The interaction between the drug and excipients included in the formulation was investigated utilizing Fourier transform infrared spectroscopy, (FTIR spectrophotometer, SHIMADZU, IRAFFINITY-1S, Japan) using the KBr pellet method. The KBr plate disc was prepared and almost 5 μL of the sample was placed on that disc and allowed to be dried in a vacuum. The samples were examined for their FTIR spectra between 4000 and 400 cm^−1^ [60]. In the current study, pure DPE and its formulated NE were analyzed for their spectra.

### 3.4. Experimental Design Study

In order to establish the current optimization study and after preliminary investigations, specific parameters were selected as independent variables such as different concentrations of surfactant (A) and oil (B). Therefore, Design-Expert version 12.0 software (Stat-Ease, USA) was implemented for developing a three-level and two-factor (32) factorial design. Each independent variable was explored at three levels to assess their influences on three responses; namely, particle size (Y1), % of in vitro drug release following 24 h (Y2), and % of hemolysis (Y3). Table 4 displayed three different levels (−1, 0, 1) that were applied for the independent variables, and their level of variation, (Y1), (Y2), and (Y3) were regarded as the dependent variable. ANOVA test provided by the software was performed to check the statistical analysis of the data such as p-value, F-value, R2, predicted R2, adjusted R2, and lack of fit. Consequently, certain model graphs were generated such as 2D Contour and 3D-response surface plot in order to demonstrate the interaction between the studied variables and responses. Moreover, mathematical modeling was conceded by using the following equation:

Y = b_0_ + b_1_A + b_2_B + b_12_AB + b_11_A^2^ + b22B^2^ (4)

where Y indicates the dependent variable, whereas b_0_ is the intercept, b_1_, b_2_, b_12_, b_11_, and b_22_ signifies the regression coefficients; A and B indicate the main factors; AB represents the interactions between main factors; A^2^ and B^2^ = polynomial terms.

### 3.5. Development of PEGylated DPE-NEs

Several NE formulations of DPE were developed using the specified amounts of constituents. 175 mg of DPE powder were added to different concentrations of arachis oil followed by vortexing. Various concentrations of surfactant (tween 80) in addition to 50 mg of PEG-DSPE were mixed with the oily phase mixture. The aqueous phase and oily phase were heated separately, after that, the aqueous phase was gradually added while homogenization at 20,000 rpm for 15 min to the oily phase using a high shear homogenizer (T 25 digital Ultra-Turrax, IKA, Staufen, Germany). The NEs formed instantly after homogenization followed by sonication for 1 min using a probe sonicator (XL-2000, Qsonica, Newtown, CT, USA) [29]. The matrix of 12 experimental formulations was assembled by (CCD) along with their observed values of response as illustrated in Table 1.

### 3.6. Characterization of DPE-NE Formulations

#### 3.6.1. Viscosity

The formulations were examined for their rheological behavior at room temperature utilizing Brookfield viscometer (DV-II + Pro, Middleboro, MA, USA) [61].

#### 3.6.2. Size and Size Distribution (PDI) Determination

DPE-NEs were analyzed for their particle size and PDI using the Zetasizer apparatus (Malvern Instruments Ltd., Worcestershire, UK). These were determined at 25 °C by assessing the dynamic light scattering at a 90° scattering angle [62].

### 3.7. Study of DPE In Vitro Release from NE Formulations

Agilent Fiber optics dissolution system (Agilent Technologies, California, USA) was utilized for estimating the percentage of DPE released from the developed NE formulations. Briefly, a glass tube closed from one side with cellophane membrane (MWCO 2000–15,000) was used to retain 2 mL of NE formulation. The tubes were utilized instead of baskets and suspended into the apparatus. A system of 750 mL PBS 7.4 was adjusted to rotate at 50 rpm and conserved at 37 ± 0.5 °C. At definite time intervals (0.25, 0.5, 1, 2 until 24 h), Samples were measured at λmax 261 nm [37]. Each experiment was performed in triplicate.

### 3.8. Hemolytic Activity

This investigation was implemented using blood obtained from rats by a syringe containing traces of heparin. Centrifugation for the collected rat blood was done at 1500× *g* for 10 min at 20 °C. Next, plasma was detached and substituted with an equivalent amount of PBS pH 7.4 followed by centrifugation, and this step was repeated three times. In a water bath adjusted at 37 °C, one mL of each NE formulation was incubated with an identical volume of erythrocyte suspension for 30 min. Afterward, the samples were subjected to 10 min centrifugation at 3000× *g* in order to separate the released hemoglobin from erythrocytes. The supernatant was diluted with PBS 7.4 and analyzed for their absorbance at λmax 550 nm. For control, erythrocytes were incubated only with PBS or mixing with 1 mL of Triton-X 100 (1%) to attain 100% hemoglobin release [37].

### 3.9. Development of Naked DPE-NE

Based on the experimental design study, one optimized NE preparation (PEG-NE) was predicted and developed. Afterward, it was necessary to develop its naked counterpart for further studies. For naked DPE-NE fabrication, the same procedure for developing PEGylated NE was followed as previously stated nevertheless without adding PEG-DSPE.

### 3.10. Zeta Potential Measurement

Zeta potential of both naked and PEGylated DPE-NE were estimated at room temperature using Zetasizer apparatus (Malvern Instruments Ltd., Worcestershire, UK). Zeta potential was measured in order to assess the NE surface charge by determining the electrophoretic mobility.

### 3.11. Determination of Serum Protein Associated on the Surface of NE Quantitatively

One milliliter from both naked and PEGylated NE formulation was incubated at 37 °C separately for 30 min with an equal volume of fresh rat serum. Thereafter, the NE formulations were passed over the Sepharose CL-4B gel column to separate the mixture from the bulk serum proteins. NEs were collected and upon using Total protein and Total Lipid Colorimetric kits, a quantitative measurement of the amount of protein attached on the NE surfaces was performed [63].

### 3.12. Studies of DPE In Vitro Release from Optimized NE (before and after Serum Incubation)

Regarding in vitro investigation, the same technique mentioned previously was monitored in order to detect the percentage of DPE released from PEG-NE and NNE preparation. The same technique was tracked for both formulations following being incubated with an equivalent amount of 10% serum [37].

### 3.13. In Vitro Cytotoxicity

The cytotoxicity of DPE and its optimized NE formulation was studied on MCF-7 and HepG2 cell lines using MTT assay [64,65]. Concisely, a 96-well plate was used for seeding 5000 cells and treating them for 48 h with adjusted concentrations of DPE, optimized PEG-NE along with the placebo NE. Subsequently, to determine the cytotoxicity, MTT was added to each well in the plat and incubated for 4 h. Then, the media was detached, followed by adding DMSO to every single well and shake for 10 min. The optical density was estimated at 570 nm [66].

### 3.14. Statistics

The values were validated as mean ± SD by applying the experiments three times at least. Student’s t-test was applied to verify the statistical differences between the groups. A one-way analysis of variance (ANOVA) followed by the least significant difference (LSD) as a post-hoc test was used to compare data from treated groups and the control group with each other. Statistical analysis was carried out using SPSS statistics software, version 9 (IBM Corporation, Armonk, NY, USA). The samples were deliberated statistically significant from each other if *p* < 0.05.

## 4. Conclusions

In the present investigation, date palm extract powder could be successfully obtained via several production phases then incorporated into nanoemulsion formulation. Response surface methodology was a valuable technique for exploring the influence of independent variables on the response values where the optimized formulation was predicted through the desirability function. The developed PEG-NE demonstrated suitable particle size, PDI, and zeta potential that represent a vital part in nanoemulsion stability intended for intravenous administration. The quantity of serum protein attached to the PEG-NE surface was considerably small and DPE in vitro release from PEG-NE was effectively extended over 24 h. Cytotoxic evaluation proposes positive activity of PEGylated NE of DPE toward MCF-7 and HepG2 cancer cell lines. The PEGylated NE of DPE of Khalas cultivar will open up a new future horizon for adjuvant therapy for cancer treatment.

## Figures and Tables

**Table 1:** Experimental design for DPE-NE formulations and their corresponding observed values of response.

Formula	Independent Variables		Response Values		
	A (g)	B (g)	Y_1_ (nm)	Y_2_ (%)	Y_3_ (%)
F1	0.2	2	225	45.4	3.5
F2	0.6	2	200	46.5	6
F3	0.68	1.5	150	62.8	6.2
F4	0.2	1	142	67.6	3.3
F5	0.11	1.5	175	61.9	2.4
F6	0.4	0.79	125	74.7	4.6
F7	0.4	1.5	170	58.2	4.8
F8	0.4	1.5	166	60.8	5
F9	0.6	1	130	70.8	5.8
F10	0.4	1.5	168	58.6	5.1
F11	0.4	2.2	235	40.4	4.7
F12	0.4	1.5	166	60	4.9

A: Surfactant concentration; B: oil concentration; Y_1_ particle size; Y_2_: In vitro release study; Y_3_: Hemolysis.

**Table 2:** Statistical analysis results of responses.

Source	Y_1_		Y_2_		Y_3_	
	*F*-Value	*p*-Value	*F*-Value	*p*-Value	*F*-Value	*p*-Value
Model	247.23	<0.0001	88.36	<0.0001	122.65	<0.0001
A	64.32	0.0002 *	14.43	0.0090 *	588.76	<0.0001 *
B	1154.38	<0.0001 *	393.75	<0.0001 *	1.60	0.2523
AB	1.38	0.2853	0.3336	0.5846	0.0000	1.0000
A²	1.14	0.3272	3.93	0.0946 *	22.16	0.0033 *
B²	12.75	0.0118 *	32.55	0.0013 *	3.16	0.1257
Lack of Fit	5.34	0.1011	3.51	0.1651	1.74	0.3299
R^2^ analysis						
R²	0.9952		0.9866		0.9903	
Adjusted R²	0.9911		0.9754		0.9822	
Predicted R²	0.9697		0.9206		0.9499	
Adequate Precision	48.0496		28.0624		34.3150	

Y_1_: particle size; Y_2_: In vitro release study; Y_3_: hemolysis; *, significant.

**Table 3:** Characterization of different DPE-NE formulations.

Formulation	Viscosity (cp)	Formulation	Viscosity (cp)
**F1**	3.42 ± 0.23	**F7**	2.65 ± 0.14
**F2**	2.83 ± 0.25	**F8**	2.67 ± 0.32
**F3**	2.70 ± 0.28	**F9**	2.98 ± 0.25
**F4**	3.14 ± 0.15	**F10**	2.85 ± 0.28
**F5**	2.97 ± 0.25	**F11**	2.38 ± 0.27
**F6**	2.49 ± 0.15	**F12**	2.92 ± 0.23

Values are stated as mean ± standard deviation (SD), *n* = 3.

**Table 4:** Objectives of the optimization process.

Independent Variable	Symbol	Level of Variation		
		−1	0	+1
Surfactant concentration (g)	A	0.2	0.4	0.6
Oil concentration (g)	B	1.5	2	2.5
Dependent variables	Symbol	Constraints		
Particle size (nm)	Y_1_	In range (140–160 nm)		
In vitro drug release (%)	Y_2_	In range (55–65%)		
Hemolysis (%)	Y_3_	Minimize		

**Table 5:** Predicted and observed experimental value of response at optimized conditions.

Response	Predicted Values	Experimental Values
Y_1_ (nm)	156.067	159.33 ± 3.4
Y_2_ (%)	65	64.3 ± 1.82
Y_3_ (%)	3.314	3.2 ± 0.15

## Data Availability

Did not report any data.
